# Descriptive epidemiology of nasopharyngeal carcinoma at Tikur Anbessa Hospital, Ethiopia

**DOI:** 10.1186/s12885-021-08311-8

**Published:** 2021-05-12

**Authors:** Elizabeth Tadesse Beyene, Siraw Girum Ketema, Assaye Nibret Alebachew, Mizan Yeshanew Saleh, Tsigereda Atumo Gebremariam

**Affiliations:** 1 Department of Otorhinolaryngology, Addis Ababa University College of Health Sciences, Addis Ababa, Ethiopia; 2 Department of Otorhinolaryngology, Yekatit 12 Hospital Medical College, Addis Ababa, Ethiopia

**Keywords:** Nasopharyngeal carcinoma, Tikur Anbessa hospital, Histopathology, Nasopharynx, Carcinoma

## Abstract

**Background:**

Nasopharyngeal cancer is distinguished from other cancers of the head and neck in its epidemiology, histopathology, clinical characteristics, and therapeutic outcome. Its unique clinico-epidemiologic pattern of the disease is an area focus for this investigation. Accordingly, the study investigated the demographic and histologic characteristics, as well as the clinical stage at presentation of nasopharyngeal carcinoma patients at Tikur Anbessa Specialized hospital.

**Methods:**

Hospital based retrospective descriptive study was conducted from September 2017 – October 2020. All biopsy proven incidental cases during the study period are included. SPSS version 26 is used for data entry and analysis.

**Result:**

A total of 318 patients with histologically confirmed nasopharyngeal carcinoma cases during the study period were included. There were 218 males and 90 females, with a male: female ratio of 2.5:1. The age of patients ranges from 13 to 81 years with a mean age of 37.8 ± 15 years. The median age at diagnosis was 38 years. Age distribution has two peaks for males, first between 30 to 39 and second 50 to 59 years of age. While the peak age of occurrence for females is in the 20–39 age range. Juvenile cases constituted 34% of the study group. The study revealed, nonkeratinizing carcinoma as the most prevalent histology at 94.3% (undifferentiated type 85.9% and differentiated keratinizing squamous cell carcinoma 8.4%) and 5.7% of the cases showed keratinizing squamous cell carcinoma. Majority of the patients, 86%, presented late with stage III and IV disease.

**Conclusion:**

Nasopharyngeal cancer is commonly found among the young and productive age group, under the age 30. Nonkeratinizing carcinoma is the predominant histopathologic variant resembling that seen in endemic areas of the world. Thus, genetic and early life environmental exposures should be well studied to identify possible risk factors in the region. Late-stage presentation at diagnosis impacts the treatment outcome of patients, thereby indicating the need for a raised index of suspicion among health professionals for early diagnosis and better prognosis of patients.

## Introduction

Nasopharyngeal carcinoma (NPC) is a malignant tumor arising from the squamous epithelial lining of nasopharynx, frequently from the area of fossa of Rosenmüller [1].

The disease is considered as one of the rare forms of cancer worldwide where only 86,500 cases of nasopharyngeal carcinoma were reported in 2012, accounting for only 0.6% of all cancers diagnosed in that year [2]. It is notable for its high incidence in selected geographic and ethnic populations. Globally the highest incidences have been observed in populations living in or originating from Southern China whereas Southeast Asia, North Africa and Inuits (Eskimos) of Canada and Alaska, all have intermediate incidence [3]. According to the 2015 population based cancer registry data of Addis Ababa, nasopharyngeal cancer was found to be the 5th commonest cancer in males and the 17th in females [4]. The incidence of NPC in men is shown to be higher than in women, with a ratio of 2–3 to 1 in both endemic and non-endemic areas of the world [5].

The histological classification of nasopharyngeal carcinoma proposed by World Health Organization (WHO), categorized tumors into three pathological types: keratinising squamous, non-keratinising (differentiated and undifferentiated subtypes), and basaloid squamous [6]. Keratinising squamous cell carcinoma is a WHO type I, which shares similar characteristic features with other head and neck squamous cell carcinomas, whereas the non-keratinising nasopharyngeal carcinoma (NK NPC), types II and III refer to non-keratinising differentiated and undifferentiated tumors, respectively [7]. The NK NPC that comprises over 95% of NPC in high-incidence areas, is correlated with raised titres of Epstein-barr virus (EBV) serology; in contrast, type I NPC is predominant in non-endemic regions, and may have an etiology distinct from that of the other two histologic types with a reduced EBV serologic titres [8, 9].

The distinct geographic and ethnic variations of NPC worldwide suggest that both environmental factors and genetic traits contribute to its development [5]. Childhood intake of preserved foods is studied as the main risk for development of NPC in endemic populations of Chinese, natives of Southeast Asia, Arabs of North Africa and natives of Artic region. These locally consumed preserved foods are assumed to share common carcinogenic substances mainly nitrosamines and EBV activating substances [10, 11]. The link between NPC and Epstein-Barr virus is well established, as patients with this malignancy were found to have a raised antibody titers against the virus [12]. A number of non-dietary environmental exposures, including domestic exposure to smoke from burning wood and incense, occupational exposure to dust, smoke and chemical fumes and, tobacco smoking, have also been suggested as risk factors for NPC [13].

Given the limited epidemiological evidence regarding NPC in Ethiopia, the present study tries to investigate the clinico-epidemiologic pattern of Nasopharyngeal Carcinoma of patients at Tikur Anbessa Specialized Hospital (TASH).

## Methods and materials

### Study area and period

The study was conducted at Tikur Anbessa Specialized Hospital (TASH), Addis Ababa, Ethiopia. Cases of biopsy-proven Nasopharyngeal Carcinoma (NPC), between September 2017 and October 2020 were investigated. TASH is the largest referral hospital in the country. It is also an institution where clinical services that are not available in other public or private institutions are rendered to the whole nation. Until recently, TASH was the only hospital in the country providing oncology service for cancer patients and carries the entire cancer burden of the country.

### Study design

Institution based retrospective review of document was carried out on histologically confirmed nasopharyngeal carcinoma patients who attended TASH during the study period.

### Study populations and sampling techniques

All incidental cases of biopsy-proven NPC during the study period were included in the study.

### Operational definition

Cases of Nasopharyngeal carcinoma were defined as; ‘A nasopharyngeal malignancy arising from the nasopharyngeal squamous epithelial lining’ as confirmed on histopathological examination of biopsy obtained from nasopharyngeal specimen.

### Data collection techniques

Medical charts of 318 NPC cases diagnosed between September 2017–October 2020 were reviewed using structured data collection questionnaire. Data collected on all patients included age, gender, residence, religion, educational background as well as clinical data of pathology results. The clinical stage at presentation was assessed according to the American Joint Committee on Cancer (AJCC) 2018 system.

### Statistical analysis and quality assurance

To ensure data quality, the collected data was checked for completeness, clarity and consistency by the investigators immediately after the data has been collected by trained data collectors. The principal investigator also closely monitored data collection and data entrance. Coding of individual questionnaires was done before data entry in to the software. The data was entered & analyzed using SPSS version 26 for statistical analysis. The descriptive statistics such as proportions, percentages, ratios, frequency distributions and appropriate cross-tabulation presentations besides measures of central tendency and measures of dispersion was used for describing data.

### Ethical considerations

Approval of this study was obtained from Institutional Review Board of College of Health Science, Addis Ababa University (Protocol No. 084/19/ENT). All methods were carried out in accordance with relevant guidelines and regulations. Informed consent was obtained from all study subjects. For subjects under 18, consent was obtained from a parent and/or legal guardian. After the diagnosis of NPC was established patients were linked to the Oncology unit, for possible radiotherapy and chemotherapy.

## Results

### Sociodemographic characteristics

In the study, a total of 318 histopathologically confirmed nasopharyngeal carcinoma (NPC) patients were included and their medical records were reviewed. Among these 228 (71.7%) are male and 90 (28.3%) of the participants are females. The youngest patient is 13 years old and the oldest 81. The mean and median ages respectively are 37.8 and 38 with a standard deviation of 15.05. The age distribution for both sexes showed a peak between the ages of 20–39 years for both sexes, which accounts for 44% of cases (Table 1).

**Table 1:** Age * gender distribution of cases of nasopharyngeal carcinoma at Tikur Anbessa Hospital from 2017 to 2020

*Age group*	*Frequency [n] (Percent)*	*Male [n] (%)*	*Female [n] (%)*
*10–19*	*38 (11.9%)*	*24 (10.5%)*	*14 (15.5%)*
***20–29***	*68 (21.4%)*	*44 (19.3%)*	*24 (26.7%)*
***30–39***	*70 (22.0%)*	*46 (20.2%)*	*24 (26.7%)*
*40–49*	*56 (17.6%)*	*36 (15.8%)*	*20 (22.2%)*
*50–59*	*54 (17.0%)*	*46 (20.2%)*	*8 (8.9%)*
*> 60*	*32 (10.1%)*	*32 (14%)*	*0 (0%)*
*Total*	*318*	*228*	*90*

The age distribution of NPC patients with respect to gender has shown different patterns of distribution. Female participants had a peak between 20 and 40 years of age, without a clear bimodal age distribution. While male participants had two peaks 30–39 years and 50–59 years of age. For both sexes the number of cases declined above the age of 60.

We observed a significant number of juvenile NPC cases, aged under 20 years (12%) and 20–29 (22%) years old (young adult). Overall, under 30 year age group represented 34% of all cases.

Geographic distribution of patients showed, 38.4% from Oromia region, followed by Amhara region 22% Addis Ababa 20.1%, Tigray 4.4%, SNNPR 6.9%, Somali 4.4% and Afar, Harari & Gambella each accounting < 2%. Sixty-two percent of the cases are from rural area of the country and 38% from the urban regions of the country.

### Clinical stage and histopathologic result

Among the total cases found in the study, 56% (*n* = 178) are stage III while 30.2% (*n* = 96) are stage IV. Stage II and I account for 9.7% (*n* = 31) and 4.1% (*n* = 13) of the cases, respectively. According to the World Health Organization (WHO) classification of histopathologic variants, non- keratinizing nasopharyngeal carcinoma is found in 94.3% of the cases studied. Of these, undifferentiated type constituted 85.9%, while differentiated nonkeratinizing carcinoma accounted 8.4% of the cases. The keratinizing type of squamous cell carcinoma was found in only 5.7% of the cases.

## Discussion

According to the data presented above, males outnumber females, with a ratio of 2.5:1, which is found to be a consistent feature across all populations in both endemic and nonendemic regions. Almost all literatures reviewed also demonstrated the incidence of nasopharyngeal carcinoma (NPC) in men being higher than women, with a ratio of 2–3:1.

The study found age distribution with similar patterns to those of intermediate incidence countries of North Africa and Southeast Asia. The peak age in the study in both male and female was found between the ages of 20–39 years (age range of 13–80) with a mean age of 37.8. Males had another peak at 50–59 years of age after which it shows a significant decline for both sexes. In an epidemiological study done in Malaysia, the incidence in both sexes rose after the age of 20–29 years and reached a plateau between 40 and 49 years. No further rise was exhibited after age 60 years [14]. In another study conducted in North African Maghreb countries, the age at diagnosis of the cases ranged from 11 to 81 years, with an apparent bimodal distribution with peaks at 20 years and 40 years [15]. The peak age differences in gender, found in this study has also shown a similar pattern to a finding of a study conducted in Ibadan Cancer Registry in Nigeria, where the overall the mean age was 41.1 years. The research also showed a peak age group of incidences for females was 20–29 and 50–59 for the males with a sharp incline in the 4th and 5th decades and rapid decline after the age of 60 [16].

The bimodality in the age distribution found in this study at Tikur Anbessa Specialized Hospital (TASH) has shown a similar pattern to that of moderate incidence regions, which might be related to the early age environmental carcinogen exposure and probable chronic Epstein-barr virus (EBV) infection although the EBV status of the cases is unknown. Hence, these findings mandate the incorporation of EBV serologic studies as a routine workup for NPC patients. On the other hand, the rapid decline in incidence after 60 years of age indicated that NPC in the study population is less likely associated with the usual risk factors related with other head and carcinomas.

Generally, NPC is uncommon in individuals under the age of 20 years, whereas in Northern Africa, an endemic area, it is found that 20% of patients are below age 30 [17]. Studies have shown notable age differences of North African and Southeast Asian countries, suggesting that it could result from a distinct combination of etiological factors. One intriguing characteristic of North African NPC is its bimodal age distribution with a secondary peak of incidence in the range of 15–25 years, not observed in Asian NPC [18]. In accordance with this finding we observed a significant number (34%) of juveline NPC cases, aged under 30 years in our study. The higher incidence of juveline NPC may reflect a possible genetic susceptibility. Thus, familial aggregation should be well investigated in the region, as family history of NPC is more likely associated with endemic forms of the disease.

Besides geographical variation, some ethnic groups also seem to have a predisposition for nasopharyngeal carcinoma. From our study participants the highest percentage, 38% of patients are from Oromia region, but this finding cannot be supported due to small sample size of the study and the Oromia region being the most populous in the country. Thus, the distribution of cases in the present study is equivalent to population size of each region.

The study also found 86.2% of patients presented with locoregional disease and late advanced clinical stage that is, stages III and IV disease. This is similar to the 89% of patients in advanced stage disease from a Kenyan study [19], and in a Tanzanian study where, 80% of patients were found to be in Stage IV [20]. Our study shows similar values for stage III and IV diseases when compared to studies from endemic areas, where stage III and IV tumor in general account for close to 80–90% of cases at presentation. These finding can be ascribed to a more aggressive course of the disease noted in the undifferentiated histopathologic variant of the disease commonly found in endemic areas of the world [21].

In addition to the geographic based age distribution, NPC shows varying histopathologic distribution among different populations. In this study, nonkeratinizing nasopharyngeal carcinoma (NK NPC) is found in 94.3% of the cases, while the keratinizing subtype was found in 5.7% of the cases. The histological findings of our study resembles that of endemic areas worldwide, with a predominance of NK NPC, especially that of undifferentiated type which accounts for 85.9%. The findings in the present study can be paralleled with a study conducted in North Africa, an endemic region, where almost all (92%) cases were undifferentiated carcinomas (UCNT) and in Indonesia which is estimated to have an intermediate incidence of NPC, is also characterized with majority of NK NPC type [22].

On the other hand, a study conducted in a similar institution, Tikur Anbessa Specialized Hospital (TASH), between 2016 and 2017 showed 70% of cases as non-keratinizing undifferentiated nasopharyngeal carcinoma while the remaining as keratinizing nasopharyngeal carcinoma [23]. In comparison, the recent growing number of predominant NK NPC in the present study reflects the recent changing trends of environmental, lifestyle and possibly genetic factors of the region, related to NPC.

In general, the histopathological variants of NPC correlate with the etiology of the disease. Most notably, the nonkeratinizing neoplasms as evidenced in this study are likely caused by chronic subclinical EBV infection similar to what has been observed in endemic regions. Since EBV carrier state is a ubiquitous condition, where more than 90% of adults worldwide have been infected with EBV, other carcinogenic cofactors, are also implicated in the etiogenesis of NPC [24]. It is therefore indicated that EBV alone is not a sufficient cause for this malignancy. Therefore, environmental exposures and/or genetic risk factors are also more likely to play a role in the pathogenesis of EBV related NK NPC in endemic regions of the world [25].

### Limitations of the study

This study used a cross-sectional design on a single institution providing only a descriptive report of the patients diagnosed at the hospital, hence it cannot represent the true distribution of NPC for Addis Ababa or Ethiopia. The EBV serologic status of the patients is unknown and thus the study provides limited evidence to explain the predominance of undifferentiated histopathologic variant in our setup, and therefore further association with EBV infection cannot be provided based on this study.

## Conclusion

According to the findings of the present study, nasopharyngeal cancer was common in the young and productive age group, which has far reaching implications in terms of their socioeconomic output. Overall, the age distribution and the pathologic findings of, nonkeratinizing carcinoma being the predominant histopathologic variant resembles that seen in endemic areas of the world. Indicating that Ethiopia is still an unexplored region regarding nasopharyngeal carcinoma, large scale studies need to be conducted to study the endemicity, as well as identify the sole etiologic factors in the region.

## Data Availability

Available upon request by contacting the corresponding author.

## Abbreviations

AJCC: American Joint Committee on Cancer
EBV: Epstein-barr virus
ENT: Ear Nose Throat
NK NPC: Non-keratinising nasopharyngeal carcinoma
NPC: Nasopharyngeal carcinoma
ORLHNS: Otorhinolaryngology Head & Neck surgery
TASH: Tikur Anbessa Specialized Hospital
UNCT: Undifferentiated carcinoma of nasopharyngeal type
WHO: World health organization
